# Radiocaesium partitioning in Japanese cedar forests following the “early” phase of Fukushima fallout redistribution

**DOI:** 10.1038/srep37618

**Published:** 2016-11-23

**Authors:** Frederic Coppin, Pierre Hurtevent, Nicolas Loffredo, Caroline Simonucci, Anthony Julien, Marc-Andre Gonze, Kenji Nanba, Yuichi Onda, Yves Thiry

**Affiliations:** 1 Institut de Radioprotection et de Sûreté Nucléaire (IRSN), PRP-ENV, SERIS, L2BT, Cadarache, France; 2 Center for Research in Isotopes and Environmental Dynamics, University of Tsukuba, 1-1-1 Tennodai, Tsukuba, Ibaraki 305-8572, Japan; 3 Institut de Radioprotection et de Sûreté Nucléaire (IRSN), PRP-DGE, SRTG, LT2S, Fontenay-aux-Roses, France; 4 Institut de Radioprotection et de Sûreté Nucléaire (IRSN), PRP-ENV, SESURE, LEREN, Cadarache, France; 5 Fukushima University. Institute of Environmental Radioactivity. 1 Kanayagawa, Fukushima City, Fukushima Prefecture, 960-1296, Japan; 6 Andra, Research and Development Division, 1-7 rue Jean-Monnet, 92298 Châtenay-Malabry, France

## Abstract

Our study focused on radiocaesium (^137^Cs) partitioning in forests, three vegetation periods after the Fukushima Daiichi nuclear power plant accident. ^137^Cs distribution in forest components (organic and mineral soil layers as well as tree compartments: stem, bark, needles, branches and roots) was measured for two Japanese cedar stand ages (17 and 33 years old). The results showed that around 85% of the initial deposit was found in the forest floor and topsoil. For the youngest stand almost 70% of the deposit is present in the forest floor, whereas for the oldest stand 50% is present in the 0–3 cm mineral soil layer. For trees, old and perennial organs (including dead and living needles and branches, litter fall and outer bark) directly exposed to the fallout remained the most contaminated. The crown concentrated 61–69% of the total tree contamination. Surprisingly the dead organs concentrated 25 ± 9% (young cedars) to 36 ± 20% (mature cedar) of the trees’ residual activity, highlighting the importance of that specific compartment in the early post-accident phase for Japanese cedar forests. Although the stem (including bark) represents the highest biomass pool, it only concentrates 3.3% and 4.6% of the initial ^137^Cs deposit for mature and young cedars, respectively.

The Fukushima Daiichi Nuclear Power Plant (FDNPP) accident in March 2011 released between 380 and 800 PBq of volatile radionuclides (iodine, tellurium and caesium) into the atmosphere, 20% of which were spread across the Japanese terrestrial environment^1^. To date, caesium radioisotopes ^137^Cs and ^134^Cs dominate in contaminated ecosystems due to their physical half-lives, (30.2 y and 2.1 y, respectively). Forest ecosystems composed of evergreen species, mainly Japanese cedar (*Cryptomeria japonica*), Japanese Cypress (*Chamaecyparis obtusa*) and deciduous species cover almost 75% of the highest contaminated area (>5 mSv y^−1^)^2^. Since the Chernobyl accident, the importance of forest radioactive contamination as a significant source of local radiation exposure has been recognised^3^, and several studies have shown a clear and persistent high level of contamination in different forest products^4^. Owing to its longevity and high standing biomass and turnover, forest vegetation can efficiently trap and recycle radioactive fallout, resulting in the potential for enhanced external and internal exposures over timescales spanning from decades to centuries^5^. After the initial deposit, the observed ^137^Cs (or ^134^Cs) contamination of the tree components is in fact the result of various processes such as interception, foliar absorption, internal translocation, root uptake and immobilisation in perennial parts^6^. Over a timescale of decades or longer, it is expected that long-lived radionuclides will be incorporated into natural elemental cycles, especially in elements such as strontium and caesium due to their chemical analogy with major nutrients (i.e. Ca and K, respectively). In multi-layered forest soils, the possible long-lasting availability of radiocaesium in particular is due to its slow vertical migration and its persistence in surface organic layers^7^,^8^,^9^. In ^137^Cs contamination dynamics, two stages can be distinguished: (1) the “early” phase lasting 4–5 years, in which the depuration processes of the tree crown predominate, characterised by the rapid redistribution of the initial deposits between trees and soil (via weathering, throughfall and litterfall), and (2) an apparent “steady state” phase characterised by the stabilisation of radioactivity transfers between soil and tree, with root uptake mainly controlling the extent of further ^137^Cs cycling^4^,^10^. Hence, a good understanding of the short- and long-term dynamics of radiocaesium in forests is essential to predicting its behaviour^11^. The type of ^137^Cs deposits (dry and/or wet) and the environmental conditions (climate, species, edaphon, topography) found near the site of the Chernobyl and Fukushima disasters are different and are likely to generate a distinctive contamination redistribution within the forest environment^12^. For instance, given the predominance of mountainous land in Fukushima, the role of the forest as a potential secondary source of contamination dissemination to other parts of the environment by runoff and erosion processes was examined^13^. In this context, monitoring contaminated forest stands is essential to providing local datasets that will allow flows between forest ecosystem compartments and driving parameters to be quantified^14^,^15^.

Many studies in the Fukushima contaminated area related to early ^137^Cs distribution in the Japanese forest ecosystems^2^,^15^,^16^,^17^,^18^,^19^. Most of these studies began immediately after the accident (main data from 2011 to 2012) and showed that for the evergreen species, interception by canopies was very high (60–90%) due to high canopy closure^20^. Depending on the tree species and on the renewal of the canopies, contamination return to the forest floor was mainly supported by litterfall and rainfall^21^,^22^. According to those observations, canopy self-decontamination is now expected to stabilise. However, there are few detailed budgets on activity partitioning for this transition period, especially those taking into account the soil-tree system as a whole.

The scientific programme launched in November 2013 aims to provide relevant parameters for modelling radiocaesium transfers in forest ecosystems at different timescales, including short to long term. Monitoring of contaminated forest stands in Japan was initiated to cover the whole forest cycle of radiocaesium over 6 years. This study describes the radiocaesium inventories taken in two Japanese cedar stands in November 2013 (i.e. three vegetative periods after the fallout) at stand scale. Its objective is to provide an initial estimate of the ^137^Cs distribution between soil and tree and also within the tree using a robust sampling method.

## Materials and Methods

### Site description

The characteristics of the monitoring sites have been detailed in previous studies^21^,^22^,^23^. Briefly, the stands are located approximately 40 km northwest of FDNPP in the Yamakiya District (Kawamata Town) and consist of two contiguous even-aged Japanese cedar stands: one young (YC, 17 years in 2013) and one more mature (MC, 33 years in 2013). The main characteristics of the forest plots are provided in Table 1.

### Field sampling and measurements

#### Trees sampling

The two stands (YC) (MC) were characterised in November 2013 by measuring the diameters at breast height (DBH, 1.30 m above the ground) within the perimeter of defined plots (n = 160 trees for MC, n = 214 trees for YC) and the densities were calculated per hectare. The normality of the DBH distributions of the two stands was statistically tested under R environment (Shapiro-Wilke test, Development Core Team^24^) and showed a normal distribution for the YC stand (p-value = 0.64) and a lognormal distribution for MC (p-value = 0.25). The populations of each stand were divided into nine DBH classes of equal size (i.e. the same number of trees in each class, n = 18 for MC, n = 24 for YC) and one tree was selected per size class to be cut and sampled. The height of each tree was measured after tree felling. The following methodology was adapted from Picard *et al*.^25^. Branches were separated from the trunk and the needles from the branches (living and dead organs). The crown compartments of the three median class trees (classes 4, 5 and 6) were all weighed (living needles and branches) to obtain the total biomass per compartment. A representative aliquot of tree compartments (i.e. organs) was then sampled: living branches (branches >1 year), twigs (current-year living branches), old living needles (cohorts 2 years old and more), current-year living needles and dead organs (branches and needles). The stems were then cut into 1 m logs to be weighed. All the logs were weighed (fw) and biometrics (diameters at each end and lengths of the 1 m logs) were measured to calculate the stem volumes according to a successive truncated cone model.

Along each stem, 2–4 cm wood discs were cut from the cutting plane to the top every 4 m for MC trees and every 2 m for YC trees. Each disc was then weighed, and the biometrics were measured according to two axes (thickness, diameter, width of outer bark, inner bark, sapwood and heartwood) and the age of each tree was checked (cutting plane of the wood disc). Then, from one quarter of each wood disc, each previously mentioned compartment was pooled for each tree. The volume of a weighed aliquot (fw: fresh weight and dw: dry weight) from each sampled organ was measured and densities were determined.

Lastly, the roots of the cut trees from the three median classes of each stand were sampled. The attached soil was removed by gently rinsing with water and they were then separated into fine (<2 mm) and coarse (>2 mm) roots. Understory roots were fully distinguished from tree roots. Samples were taken until a depth of 40–50 cm below the litter layer which contains more than 80% of the fine roots^26^.

All fresh weights were determined just after sampling and all samples were dried at 70 °C until constant weight (dw) was reached. The dry samples were then homogenised and crushed in a blender (Waring^®^ CB-15T) and stored in sealed bags for further analyses.

#### Soil sampling

Five-layer sampling was performed (n = 7/plot): litter layer, organic fragmented layer and three soil layers according to depth (soil 0–3 cm, soil 3–8 cm and soil 8–20 cm). The organic humified layer was not clearly identified, and was therefore not distinguished from the organic fragmented layer. The litter and organic fragmented layers were all sampled using a 545 cm^2^ aluminium frame (19.4 × 28.1 cm). This sampling method was repeated twice for each sampling location (total sampling area 1,090 cm^2^), then the litter and organic fragmented materials were kept in sealed plastic bags to be weighed. Below the organic layers, a pit was dug to sample an undisturbed soil profile and two open plastic boxes (720 cm^3^ volume, 9 × 4 × 20 cm each) were placed in the soil, with the top of the box corresponding to the top of soil 0–3 cm. When the boxes were removed the soil 0–3 cm, soil 3–8 cm and soil 8–20 cm heights were measured, then carefully separated, weighed (fw) and homogenised for each corresponding layer. The collected samples were dried at 105 °C until constant weight was reached. The fresh and dry surface densities (kg m^−2^) for the five layers and the volumetric densities (kg dm^−3^) for the three soil layers were determined. The dry samples were crushed with a mortar for further analyses.

#### Radiocaesium measurements

An aliquot of the crushed samples was transferred into polystyrene containers for gamma spectrometry analyses. One part of the radiocaesium measurements (^134^Cs and ^137^Cs) was taken at Fukushima University while the other part was taken at the Institute for Radiological Protection and Nuclear Safety (IRSN) in Cadarache. At Fukushima University, the samples were packed into 100-mL polystyrene containers (U-8 geometry) and analysed using an ultra-pure germanium gamma spectrometer (ORTEC GEM40–76, P type, relative efficiency 44%). At IRSN, the samples were packed into 17-mL polystyrene containers (K geometry) and analysed using an ultra-pure germanium gamma spectrometer (Canberra EGPC 42-190-R, P type, relative efficiency 41%). The accuracy of the ^137^Cs measurement intercomparisons carried out on 30% of samples was less than 5%, which is below the measurement uncertainties (6–8%). All the activities were decay corrected to the 11^th^ of March 2011.

#### Calculation of the biomass and its distribution in tree compartments

**Figure 1:**
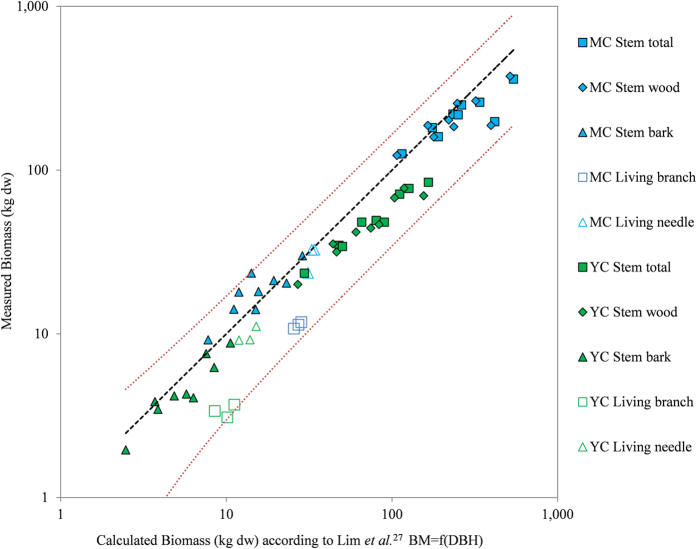
Comparison of the measured dry biomass with the dry biomass calculated for each tree DBH using Lim *et al*.^27^ allometric equations (Weight = a*DBH^b^).

To obtain the total biomass at stand scale, the allometric equation and parameters published by Lim *et al*.^27^ for living organs were applied to all trees in the plots after checking the correlation between the predicted values and the measured biomass of the organs of the cut trees (stem total (n = 18), foliage and branches (n = 6) and the values for the bark (n = 18) and the stem wood (n = 18) based on their respective biomass distribution measured in wood discs) (Fig. 1).

The distributions of the stem compartment (outer bark, inner bark, sapwood and heartwood) biomasses were calculated using the successive truncated cone model (circular section), calibrated by specific biometrics on wood discs. These distributions were validated by comparing aggregated modelled biomasses of stem organs with measured stem biomasses (mean difference of 5%, maximum difference of 12%) (Fig. 1).

The biomasses of the root compartments were calculated using the allometric equations reported by Lim *et al*.^27^ for total biomass (W = a + bD^2^, where W is the dry biomass in g, D is the DBH in cm, a = 1,198.17 and b = 85.044), and the distribution between fine (<2 mm) and coarse (>2 mm) roots was derived from Fujimaki *et al*.^26^ data according to the age of the stand.

With regard to the dead organs (branches and needles) of the MC stand, the allometric equations published by Yoshida and Hijii^28^ were used (W = a (D^2^)^b^, where W is the dry biomass in g, D is the DBH in cm, a = 95.7 and b = 0.728 for dead needles, a = 16.1 and b = 0.970 for dead branches). The parameters of these allometric equations were obtained from a specific stand with dendrometric characteristics that are very similar to those of the MC stand. For the dead organs (branches + needles: dead material in-crown) of the YC stand, exhibiting different dendrometric characteristics, we used a regression model, plotting the log-transformed literature values reported for younger stands in the literature^28^,^29^,^30^ against their log-transformed height (H) values (N = 9). A mean value regression curve was obtained from fitted value regression^31^: dead material in-crown = aH^b^, where a = 0.0361, b = 2.2, R^2^ = 0.80, F-statistic^24^: 28 on 1 and 7 DF, p-value = 0.001. The relative abundance of dead needles versus dead branches (1.4) was calculated according to Yoshida and Hijii^28^. Details of the dead biomass calculation are given in Supplementary information online.

#### Calculation of the radiocaesium inventory in the forest ecosystem compartments

The ^137^Cs inventories (Bq m^−2^) in the compartments of each forest plot were calculated by multiplying the measured ^137^Cs concentrations (Bq kg_dw_^−1^) in the different tree organs or soil layers by their corresponding biomass or surface density (kg_dw_ m^−2^) respectively. For tree compartments, ^137^Cs concentrations were not found to be dependent upon DBH (data not shown), and the mean values of the ^137^Cs concentrations were used.

Only ^137^Cs data are presented in this paper because the average ratio between ^137^Cs and ^134^Cs measurements was calculated, which did not differ from 1 at the date of reference (1.06 ± 0.15, data series not shown) and corresponded well to previous reported values^1^,^32^,^33^. The delta method was used to allow for the propagation of error in the calculation chains^34^.

## Results and Discussion

### Distribution of tree biomass and soil surface densities

The obtained tree biomass and soil surface densities (kg_dw_ m^−2^) are reported in Table 2. As expected for tree compartments, the stem is the main one representing 69–71% of the above-ground biomass for YC and MC respectively. The living foliage (current and old needles) and living branch biomasses respectively represent 9% and 8% for MC and 12% and 9% for YC. This slight difference between stands is due to the growth dynamics associated with recent canopy closure for YC. Biomass and heartwood contributions are naturally different between both stands (MC > YC), and the contribution of the bark compartment is evidently lower for MC. It is interesting to note that the biomass of dead needles and dead branches remaining in the crown is high, accounting for almost 43% (MC) and 22% (YC) of the biomass of the respective living organs (dead needles: 4% and 3%; dead branches: 4% and 2% of the above-ground biomass for MC and YC, respectively). The roots represented 20% and 23% of the total tree biomass for MC and YC respectively. For the soil layers, the surface density of the organic layers is 60% higher in the YC stand compared with the MC stand (2.5 compared with 1.5 kg_dw_ m^−2^), whereas the mineral soils have the same densities. This difference between stands is probably correlated to annual litterfall amounts as suggested by the stands’ in-crown dead biomass turnover of stands (0.35 y^−1^ and 0.88 y^−1^ for MC and YC, respectively).

### ^137^Cs massic activities in the forest compartments

**Figure 2:**
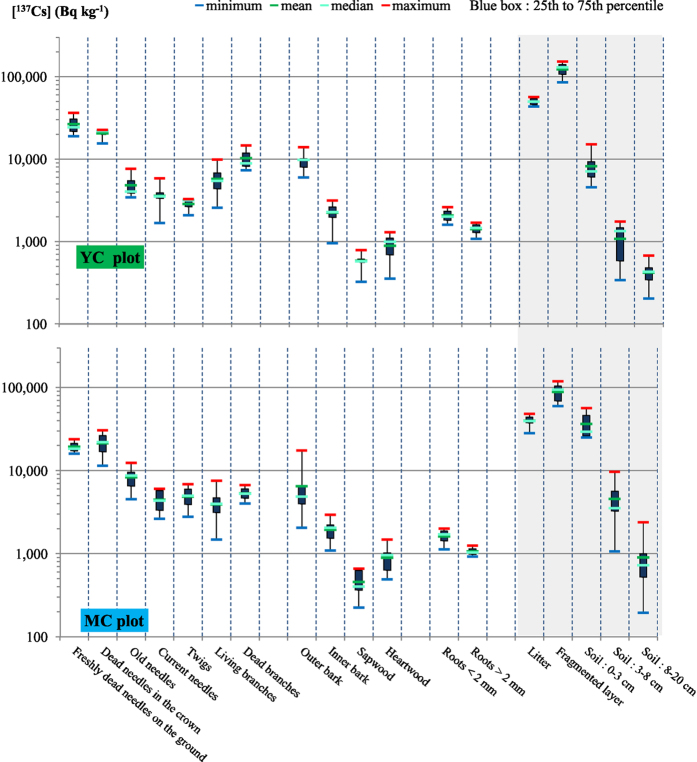
Observed ranges of ^137^Cs activities in trees sampled organs and forest floor layers.

The mean values of ^137^Cs activities (±1SD) for the various forest compartments are tabulated in Table 3 and displayed in Fig. 2. In a first approximation, no obvious difference in contamination distribution was detected between MC and YC stands. As regards trees organs, Fig. 2 depicts the ranges of measured values and shows that three vegetation periods after the Fukushima fallout the organs directly exposed to ^137^Cs deposits, even partially, (litterfall, dead needles, old needles, living branches, dead branches and outer bark) still exhibit the highest concentrations in both plots. The tree crown remains the most contaminated compartment compared with other tree parts (i.e. stem and roots) suggesting that the early phase, involving predominant crown depuration processes, is not complete three vegetation periods after the accident and may even persist for several years, with litterfall being a major source of ^137^Cs transfer. In our context, the contamination of the other organs not directly exposed to the initial fallout or which grew after the fallout (i.e. needles young, twigs, inner bark, sapwood, heartwood and roots) can be considered a consequence of internal transfers which were shown to be very efficient for ^137^Cs^35^,^36^ or processes that diffuse the contamination within/down the canopy (throughfall, rain splash, dripping, etc.). It is therefore possible than ^137^Cs redistribution in cedar trees through internal transfers is not yet stabilised.

As already observed in other monitoring studies for Japanese cedar^17^,^18^ both heartwood and sapwood compartments are contaminated. Although the contamination levels are of the same order of magnitude, a positive gradient from external (sapwood) rings to internal rings (heartwood) can be observed as a trend, implying that Cs^+^ cation has a strong radial mobility^37^. This positive gradient of concentration from sapwood to the pith was previously observed for potassium and caesium for Japanese cedar^38^,^39^,^40^ and their accumulation in heartwood was associated with the formation of these tissues^39^. This peculiarity was not observed in post-Chernobyl studies on pine trees or birches, however, for which the reverse trend gradient prevailed^6^,^41^, i.e. an increasing concentration from the pith to the external rings.

The similar ^137^Cs root concentrations found in the stands, but different ^137^Cs vertical distributions in the soil profile, and the ^137^Cs inner bark concentrations suggest a potential ^137^Cs downward flow following foliar uptake through phloemic pathways. This assumption was corroborated by the fact that root ^137^Cs concentration is very similar at the two sites, even though ^137^Cs vertical distribution is different. While the foliar incorporation pathway is most probably predominant at this stage^35^,^42^,^43^,^44^ our results do not make it possible to assess the relative contributions of each pathway, foliar or root uptake, and only the ^137^Cs/analogue (K) or stable isotope (^133^Cs) ratios could be used to address this issue in future studies.

In the soil compartments, the fragmented layer was the most contaminated compartment in the investigated soil profile, at 89 and 124 kBq kg_dw_^−1^ for MC and YC stands, respectively (Table 3 and Fig. 2). The litter layer was less contaminated (40–50 kBq kg_dw_^−1^) than the fragmented layer suggesting a decrease in ^137^Cs concentration within litterfall over time, as already observed^22^. This assumption is also supported by comparing litter and dead needles (20 kBq kg_dry_^−1^) activities. Although the activities are in the same order of magnitude, dead needles is less contaminated than the litter, suggesting that litter with a higher activity than dead needles is present in the litter layer. For the mineral soil layers (soil 0–3 cm, soil 3–8 cm and soil 8–20 cm), the ^137^Cs activities classically decreased with the sampling depth (Table 3 and Fig. 2) as has already been observed in other studies^45^,^46^. However, a significant difference was observed between the two plots. In the MC plot, litter layer and the first soil layer (soil 0–3 cm) exhibited the same ^137^Cs activities (37–40 kBq kg_dw_^−1^) whereas in the YC plot the ^137^Cs activity of soil 0–3 cm (8 kBq kg_dw_^−1^) was very low compared with the litter layer activity (50 kBq kg_dw_^−1^). Kato *et al*.^22^ reported that the initial deposit on the forest floor was lower in YC than in MC. They observed that contamination of the forest floor a few months after the accident (July 2011) was two times higher for MC than YC. This observation might be explained by a higher initial interception of fallout in the YC stand characterised by a higher LAI value (Table 1). As a result, for YC, the ^137^Cs concentration profiles, as depicted in Table 3, seem to reflect a delayed downward transfer of ^137^Cs from the crown to the forest floor with a lower massic activity for soil 0–3 cm compared with MC.

### Inventories of ^137^Cs in the forest compartments

**Figure 3:**
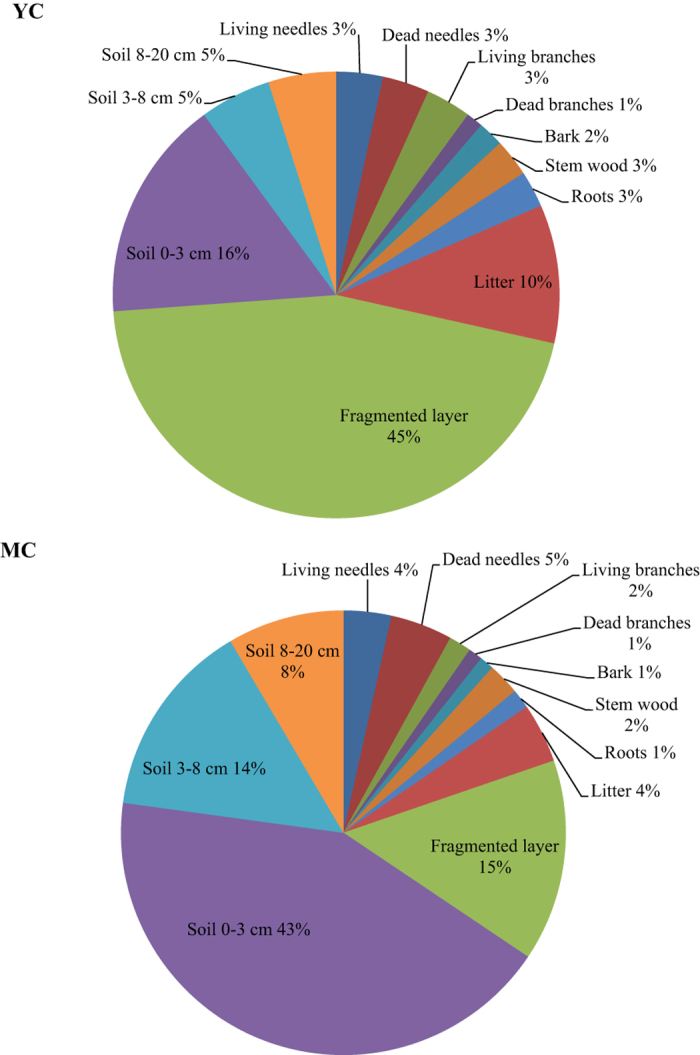
^137^Cs distribution in the forest compartments (%) of the forest plot YC and MC.

The ^137^Cs inventories (kBq m^−2^) and corresponding relative distributions for each forest compartment are tabulated in Table 4 and depicted in Fig. 3. When considering the whole forest, the total ^137^Cs inventories were estimated at 568 ± 92 and 432 ± 98 kBq m^−2^ for the MC stand and YC stand, respectively. With consideration of uncertainties resulting from spatial variability and in the estimation of the stand-level inventories in each compartment, these results are in close correlation with the total deposit of 442 ± 30 kBq m^−2^ already calculated for this area^23^ using airborne monitoring data in May 2011^47^.

#### Tree compartments

**Figure 4:**
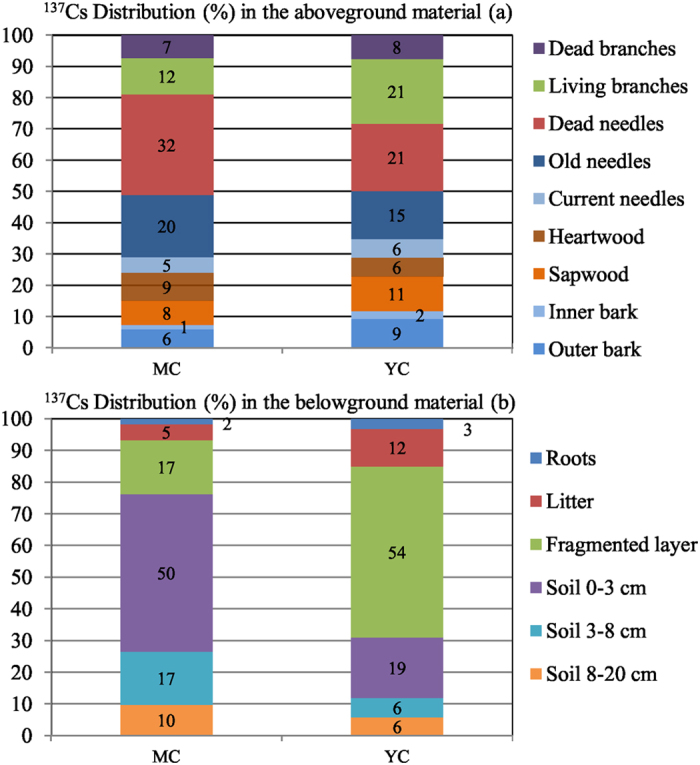
^137^Cs relative contribution in the Japanese cedars aboveground parts of each stands (**a**) and in the belowground material (**b**).

The ^137^Cs inventories in the above-ground parts of MC and YC stands (80 and 68 kBq m^−2^, respectively) contribute to 14% and 16% of the total ^137^Cs inventories respectively (Table 4 and Fig. 4). Not including the dead material (i.e. dead needles and dead branches), the ^137^Cs inventory in the above-ground parts of stands represents around 9% and 11% for MC and YC stands, respectively. These values are consistent with those obtained in 2013 by Kajimoto *et al*.^17^ for the above-ground parts of four Japanese cedar monitored stands (discarding dead material), with the respective ^137^Cs fallout deposit values released from Komatsu *et al*.^12^ the contributions varied from 6–11% of the total amount of ^137^Cs deposit, highlighting the importance of the dead material present in the crowns, at least during the early post-accident phase. Similar ranges of values for the same time period after ^137^Cs release were also published for forests contaminated by the Chernobyl accident (6–8%), but without distinguishing evergreen and deciduous species^48^,^49^.

The crown (foliage and branches) contributes to 76% (MC) and 71% (YC) of the above-ground inventories (Fig. 4). The contributions of current needles to crown inventories (5–6%) are the same for both stands (Fig. 4). Differences between stands are mostly due to respective ^137^Cs concentrations (vs. biomass). When focusing on dead materials, these compartments represent 17% (YC) to 31% (MC) of the total crown biomass (dw). However, contamination of these compartments alone contributes to more than 40% of the crown ^137^Cs inventories: 52% of crown ^137^Cs stocks for MC and 41% for YC. The difference of ^137^Cs inventory/biomass ratios for dead materials between stands (2.4 and 1.7 for YC and MC, respectively) suggests a higher interception of the FDNPP releases in YC stand. It can be explained by a higher crown biomass and LAI (Table 1) in the YC stand in parallel with its tree density, recent canopy closure and related growth dynamic. Yoshida and Hijii^28^, and references therein, reported that for Japanese cedar the turnover of crown dead material varied within a range of 1 order of magnitude from 0.17 y^−1^ to 1.9 y^−1^. They also reported that although needle lifespan physiologically reaches 6 years for Japanese cedar, the dynamic of litterfall was mostly affected by physical factors (snow, wind). The litterfall biomass amounts (g m^−2^) recorded for the stands of this study^22^,^50^ from May 2011 to May 2012 and from October 2011 to October 2012, respectively, reached a factor of 1.5 (YC) to 1.9 (MC) between the two recording periods and accurately depict the variability of this flow. Litterfall dynamics is a sensitive parameter for modeling^137^Cs redistribution during the early post-accident phase, and its inter-annual variability, especially for Japanese cedar stands, remains an issue that should be addressed, at least until foliage renewal becomes effective.

The stem wood compartments (sapwood and heartwood) account for the majority of stand biomass (from 69% for YC to 71% for MC for the above-ground biomass dw, including dead materials), but related ^137^Cs inventories contribute to only 15% of the above-ground ^137^Cs inventories. The relative contributions of heartwood and sapwood to stem ^137^Cs inventories differ according to the stand, since heartwood’s contribution to stem biomass is naturally smaller for younger forest stands. When comparing our ^137^Cs inventory data with that obtained by Kajimoto *et al*.^17^ for the same compartments sampled in September 2013 in Japanese cedar stands exhibiting the same biomass distribution, the same trends, i.e. a major contribution of crown contamination, can be observed. The ^137^Cs distribution between crowns (living needles and branches) and stems (sapwood, heartwood, outer bark and inner bark) ranges from 76–93% to 7–24%, respectively.

Although roots constitute 20% (MC) and 23% (YC) of stand biomass, this compartment accounted for only 9% and 14% of the ^137^Cs inventory in the trees (1% and 3% of the total ^137^Cs inventory) for MC and YC, respectively.

#### Soil compartments

The soil compartments are the largest pool of ^137^Cs in the forest ecosystem (85% and 82% for MC and YC, respectively, 86% and 84% if root compartments are included) and there was some difference between the two plots. Despite the fact that the fragmented layer has the highest mass concentrations in both stands (Table 2), its ^137^Cs inventory in MC is much smaller than that of the soil 0–3 cm layer (Table 4). This result is consistent with a higher ^137^Cs initial deposit on the MC forest floor^22^ and a thinner organic layer for the MC plot (1.5 vs. 2.5 kg_dry_ m^−2^, Table 2). The difference in organic layer thickness between the two plots generates a lower ^137^Cs residence time in the MC plot, either resulting from organic material degradation or leaching, as has already been mentioned^48^. In addition, the higher initial ^137^Cs interception for YC generated a delayed downward transfer of ^137^Cs from the crown to the forest floor, and also from the organic layer to soil 0–3 cm. In the next decade, the importance of forest floor thickness is expected to play a key role in further ^137^Cs migration and resulting vertical distribution as evidenced in post-Chernobyl studies^7^,^51^,^52^. Focusing on the soil ^137^Cs profile, we observed that for the MC plot, 22% of the ^137^Cs soil inventory is present in the organic layer and around 68% in the 0–8 cm mineral layers (Fig. 4). These results correlate with those obtained by Fujii *et al*.^53^ in 2012 for stands of the same age (18–26% in the organic layer and 68–69% in the 0–5 cm mineral layers). As shown in Fig. 4 for the YC plot, due to the higher initial interception generating a delay in ^137^Cs transfer from the crown to the soil compared to MC plot, the ^137^Cs profile (68% in the organic layer, 26% in the 0–8 cm mineral layers) is quite similar to the distribution observed immediately after the accident^53^. Although the main stock of ^137^Cs is concentrated in the upper forest floor layers, we observed that ^137^Cs has migrated deeper than 8 cm, with 10% and 6% of the soil ^137^Cs budget measured in the 8–20 cm layer for MC and YC respectively (Fig. 4). In forest soils ^137^Cs can be highly concentrated in organic layers, or immobilised in the first surface soil layers^46^,^54^. However, a small fraction of ^137^Cs is highly mobile and could quickly migrate to deeper mineral layers. These last authors concluded that the high content of organic matter vs. clay mineral compared with other land soils could be responsible for this higher mobility. This higher availability in organic soils has also already been observed^55^,^56^. However, the impact of organic matter content on ^137^Cs behaviour depends on the concentration and nature of the clay mineral. In our soils, only kaolinite was found in the mineral layers (Table 1), a clay mineral known to sorb caesium to a lesser degree than illite or vermiculite^57^. This observation corresponds with the high mobility of part of the ^137^Cs in our forest soils.

Even if a difference was observed between the two plots, the ^137^Cs remains concentrated in the organic layer or the upper mineral soil layer. Higher levels of ^137^Cs contamination in the organic layer compared with the mineral layers were also observed after the Chernobyl disaster, even a long time after the accident^9^,^51^.

## Conclusion

This monitoring, carried out three vegetation periods after the FDNPP accident, produced a detailed dataset on ^137^Cs concentrations and inventories in forests (trees and soil components). The sampling strategy (9 trees and 7 forest floor locations) was implemented to integrate the spatial variability occurring in two forest stands of different ages. Our results showed that the forest floor exhibited around 85% of the ^137^Cs inventories and is the major contamination reservoir three years after the accident. For the young stand (YC) with the highest initial interception, major contamination is present in the organic layer whereas on the MC stand the main contamination is present in the first mineral layer (0–3 cm). These results suggest that a delay in vertical ^137^Cs migration in YC forest soil occurred due to (1) a higher initial interception and therefore a lower initial deposit on the forest floor and (2) a thicker organic layer. Focusing on the trees, the crowns remained the most contaminated compartment (around 70% of the above-ground part ^137^Cs). Interestingly, the dead organs (needles and branches) range from 40–50% of the residual ^137^Cs in the crown. That fraction, which can be a higher range of estimation due to uncertainty biomass, is expected to greatly vary according to local forest conditions and management. Even though stems represent the largest biomass pool (70%), their contamination is low at less than 1Bq g^−1^. The compartments directly exposed to the fallout remain the most contaminated and a potential source of further ^137^Cs transfer into the soil in the next vegetation periods. As scheduled in the scientific project running up until 2019, only plot monitoring over a period of years will make it possible to characterise the transition to the apparent steady state phase, where root uptake is expected to become the main process for tree contamination. In order to gain a good understanding and allow better modelling of the ^137^Cs dynamics in Japanese cedar forests, it is also crucial to integrate biomass dynamics, translocation phenomenon and bioavailability of ^137^Cs in the soil as a whole.

## Additional Information

**How to cite this article**: Coppin, F. *et al*. Radiocaesium partitioning in Japanese cedar forests following the “early” phase of Fukushima fallout redistribution. *Sci. Rep.* **6**, 37618; doi: 10.1038/srep37618 (2016).

**Publisher’s note:** Springer Nature remains neutral with regard to jurisdictional claims in published maps and institutional affiliations.

## Supplementary Material

Supplementary Information

## Figures and Tables

**Table 1:** Main characteristics of sampling plots.

Site	Altitude, m	550	
	Annual precipitation (mm)	1,248^a^	
	Mean daily temperature (min, max) (°C)	12.4 (−9.7/37)^a^	
	Nature of soil^58^	Andosol	
	^137^Cs contamination deposit (kBq m^−2^)	442 ± 30^b^	
Plots	Japanese cedar	MC	YC
	Age of settlement (in 2013)	33	17
	Stand area (m^2^)	~2,900	~2,600
	Settlement density (tree ha^−1^)	~800	~2,400
	Mean slope (%)	20	40
	Mean tree height (m)	22.5	14
	Mean/Median DBH (m)	0.317/0.310	0.187/0.188
	Basal area (m^2^ ha^−1^)	64.3	68.4
	Leaf Area Index (LAI) (m^2^ m^−2^)^c^	4.2	10.3
	Last thinning	2004	2009
	Last pruning	1999	2010
	Mean crown height (m)	9.2	7.6
	Mean of dead branches height (m)	7.2	6.6
	Soil		
	Mean pH of organic layers	5.9 ± 0.4	6.2 ± 0.5
	Mean pH of mineral layers	5.7 ± 0.4	5.7 ± 0.5
	Main minerals in mineral layers	Quartz, anorthite, magnetite, kaolinite	
	C_org_ (%) organic layers	35 ± 8	33 ± 7
	C_org_ (%) mineral layers	11 ± 5	10 ± 3

^a^from JMA, Nihonmatsu station, year 2013,

^b^from Loffredo *et al*.^23^,

^c^raw values without clumping correction measured with a Li-Cor Plant Canopy Analyzer LAI2000.

**Table 2:** Mean values of biomass and forest floor surface densities (kg_dw_ m^−2^) of the MC and YC stands components (n = 7 for soil layers).

	plotkg_dw_ m^−2^		MC	YC
			Mean ± sd	Mean ± sd
Tree components	Needles	Living needles^a^	2.77 ± 0.96	3.31 ± 1.16
		Dead needles^b^	1.19 ± 0.34	0.71 ± 0.13
	Branches	Living branches^a^	2.34 ± 0.94	2.44 ± 0.97
		Dead branches^b^	1.09 ± 0.42	0.50 ± 0.09
	Bark	Total^a^	1.29 ± 0.37	1.38 ± 0.39
		Outer bark	0.73 ± 0.29	0.64 ± 0.26
		Inner bark	0.56 ± 0.23	0.73 ± 0.29
	Stem wood	Total^a^	20.89 ± 7.41	18.15 ± 6.73
		Sapwood	12.98 ± 6.25	13.57 ± 6.32
		Heartwood	7.91 ± 3.98	4.59 ± 2.32
	Roots	Total^a^	7.20 ± 2.82	7.72 ± 2.94
		<2 mm^c^	0.44 ± 0.41	0.64 ± 0.56
		>2 mm^c^	6.76 ± 2.68	7.08 ± 2.74
Forest floor	Organic layers	Litter	0.60 ± 0.26	0.88 ± 0.29
		Fragmented layer	0.93 ± 0.33	1.58 ± 0.52
	Mineral soil layers	Soil 0–3 cm	6.96 ± 1.60	8.41 ± 1.47
		Soil 3–8 cm	19.17 ± 3.13	20.6 ± 2.54
		Soil 8–20 cm	52.36 ± 3.75	49.95 ± 3.08

^a^Calculated from Lim *et al*.^27^.

^b^Calculated from literature^28^,^29^,^30^.

^c^Derived from Fujimaki *et al*.^26^.

**Table 3:** Mean values of ^137^Cs concentrations measured in the samples (kBq kg_dw_^−1^) (activities corrected to 2011/03/11, n = 9 for trees organs except n = 3 for Roots, n = 7 for soil layers).

	plotkBq kg_dw_^−1^		MC	YC
			Mean ± sd	Mean ± sd
Tree components	Needles	Living needles	7.12 ± 0.40	4.41 ± 0.40
		Old needles (>1 year)	8.40 ± 2.90	4.84 ± 1.39
		Current needles (<1 year)	4.48 ± 1.24	3.59 ± 1.18
		Dead needles	21.52 ± 9.67	20.63 ± 3.42
	Branches	Living branches	3.94 ± 1.75	5.81 ± 2.34
		Dead branches	5.37 ± 1.35	10.39 ± 3.88
	Bark	Total	4.57 ± 2.69	5.83 ± 1.32
		Outer bark	6.56 ± 4.69	9.88 ± 2.62
		Inner bark	1.96 ± 0.59	2.26 ± 0.68
	Stem wood	Total	0.63 ± 0.43	0.64 ± 0.43
		Sapwood	0.47 ± 0.13	0.56 ± 0.13
		Heartwood	0.90 ± 0.32	0.90 ± 0.30
	Roots	Total	1.11 ± 0.04	1.48 ± 0.06
		<2 mm	1.63 ± 0.45	2.09 ± 0.52
		>2 mm	1.08 ± 0.17	1.42 ± 0.31
Forest floor	Organic layers	Litter	39.99 ± 6.57	50.26 ± 5.21
		Fragmented layer	88.92 ± 23.03	123.69 ± 25.62
	Mineral soil	Soil 0–3 cm	36.66 ± 13.77	8.26 ± 3.56
		Soil 3–8 cm	4.60 ± 2.93	1.08 ± 0.57
		Soil 8–20 cm	0.91 ± 0.73	0.42 ± 0.16

**Table 4:** ^137^Cs inventories (kBq m^−2^) and distribution (%) in the forest compartments (activities corrected to 2011/03/11).

	Stand		MC			YC		
			kBq m^−2^	% total ^137^Cs inventory	% ^137^Cs by considering only tree	kBq m^−2^	% total ^137^Cs inventory	% ^137^Cs by considering only tree or forest floor
Tree compartments	Needles	Living needles	19.7 ± 6.9	3.5 ± 1.3	22.6 ± 9.5	14.6 ± 5.3	3.4 ± 1.4	18.3 ± 7.4
		Dead needles	25.6 ± 13.7	4.5 ± 2.5	29.2 ± 17.0	14.6 ± 3.7	3.4 ± 1.1	18.4 ± 5.7
	Branches	Living branches	9.2 ± 5.5	1.6 ± 1.0	10.5 ± 6.8	14.2 ± 8.0	3.3 ± 2.0	17.8 ± 10.6
		Dead branches	5.8 ± 2.7	1.0 ± 0.5	6.7 ± 3.4	5.2 ± 2.2	1.2 ± 0.6	6.5 ± 3.0
	Bark	Total	5.9 ± 3.9	1.0 ± 0.7	6.7 ± 4.7	8.0 ± 2.9	1.9 ± 0.8	10.1 ± 4.1
		Outer bark	4.8 ± 3.9	0.8 ± 0.7	5.5 ± 4.7	6.4 ± 3.1	1.5 ± 0.8	8.0 ± 4.1
		Inner bark	1.1 ± 0.5	0.2 ± 0.1	1.2 ± 0.7	1.7 ± 0.8	0.4 ± 0.2	2.1 ± 1.1
	Stemwood	Total	13.2 ± 10.2	2.3 ± 1.8	15.1 ± 12.2	11.7 ± 8.9	2.7 ± 2.1	14.7 ± 11.5
		Sapwood	6.1 ± 3.4	1.1 ± 0.6	7.0 ± 4.2	7.6 ± 3.9	1.8 ± 1.0	9.5 ± 5.2
		Heartwood	7.1 ± 4.4	1.3 ± 0.8	8.1 ± 5.3	4.1 ± 2.5	1.0 ± 0.6	5.2 ± 3.3
	Roots	Total	8.0 ± 3.1	1.4 ± 0.6	9.1 ± 4.2	11.4 ± 4.4	2.6 ± 1.2	14.3 ± 6.1
		<2 mm	0.7 ± 0.7	0.1 ± 0.1	0.8 ± 0.8	1.3 ± 1.2	0.3 ± 0.3	1.7 ± 1.6
		>2 mm	7.3 ± 3.1	1.3 ± 0.6	8.3 ± 4.0	10.1 ± 4.5	2.3 ± 1.2	12.6 ± 6.1
					% ^137^Cs by considering only soil and forest floor			% ^137^Cs by considering only soil and forest floor
Soil	Organic layers	Litter	24.5 ± 11.7	4.3 ± 2.2	5.1 ± 2.6	43.4 ± 12.8	10.0 ± 3.7	12.3 ± 5.0
		Fragmented layer	83.3 ± 38.3	14.7 ± 7.2	17.4 ± 8.6	195.8 ± 87.3	45.3 ± 22.7	55.6 ± 29.1
	Mineral soil	Soil 0–3 cm	242.6 ± 57.5	42.7 ± 12.3	50.5 ± 15.2	69.5 ± 37.3	16.1 ± 9.4	19.7 ± 11.9
		Soil 3–8 cm	81.5 ± 38.3	14.4 ± 7.1	17.0 ± 8.6	22.3 ± 11.7	5.2 ± 3.0	6.3 ± 3.8
		Soil 8–20 cm	48.1 ± 40.5	8.5 ± 7.3	10.0 ± 8.6	21.1 ± 7.5	4.9 ± 2.1	6.0 ± 2.7
	Above ground		79.5 ± 19.8	14.0 ± 3.6		68.3 ± 14.1	15.8 ± 4.0	
	Below ground*		488.0 ± 89.6	86.0 ± 17.6		363.6 ± 96.9	84.2 ± 25.1	
	Total stand		567.5 ± 91.6	100		431.9 ± 97.9	100	

^*^including roots.
