# Supplementary material for: Radiocaesium partitioning in Japanese cedar forests following the “early” phase of Fukushima fallout redistribution
Source: Sci Rep. 2016 Nov 23;6:37618. doi: 10.1038/srep37618 (PMC5120304; doi:10.1038/srep37618)
Supplement: Supplementary Information [file srep37618-s1.doc]

**Radiocaesium partitioning in Japanese cedar forests following the “early” phase of Fukushima fallout redistribution**

**Authors :** Frederic Coppin*^(1)^, Pierre Hurtevent^(1)^, Nicolas Loffredo^(2)^, Caroline Simonucci^(3)^, Anthony Julien^(3)^, Marc-Andre Gonze^(4)^, Kenji Nanba^(5)^, Yuichi Onda^(2)^ and Yves Thiry^(6^**)**

**Supporting Information**

**Available Literature**

We detail hereafter the way the values of the Dead Materials in-Crown (DMIC: dead branches + dead needles) attached to the stems were obtained. Several authors have reported the values of DMIC amount - for Japanese cedars stands^1-7^ as shown in Supplementary Table 1 associated with their respective stands characteristics. These values were used for further estimation and calculation.

Supplementary Table 1: Reported DMIC values for Cedar forests and their associated stands characteristics

| Reference | Dead Material In Crown  (DMIC, t/ha) | Mean Height  (H, m) | Age  (Y, years) | Density  (d, tree/ha) | Mean DBH  (DBH, cm) |
| --- | --- | --- | --- | --- | --- |
| Saito and Shidei^1^ | 4.3 | 8.8 | 11 | 5600 | 8.9 |
| Katsuno *et al.*^2^ | 7.4 | 12.9 | 21 | 3500 | 13.6 |
| Miyaura and Hozumi^3^ | 3.6 | 8.5 | 16 | 2160 | 11.6 |
| Tange *et al.*^4^ | 6.1 | 9.6 | 19 | 6600 | 10.0 |
| Tange *et al.*^4^ | 7.0 | 10.3 | 19 | 9700 | 9.5 |
| Tange *et al.*^4^ | 6.9 | 12.2 | 19 | 3280 | 14.0 |
| Katagiri *et al.*^5^ | 9.5 | 11.9 | 24 | 2543 | 15.7 |
| Kaneko *et al.*^6^ | 5.5 | 13.2 | 29 | 2425 | 19.0 |
| Yoshida and Hijii^7^ | 34.1 | 20 | 33 | 2000 | 23.3 |

**DMIC calculation for Young Cedar (YC) stand**

We plotted the log values of the stands variables (n=9) against each other to obtain the respective coefficient of the determination . These values are given in Supplementary Table 2.

Supplementary Table 2: Coefficients Determination among the stands variables

| R² | Log DMIC | Log H | Log Y | Log DBH | Log d |
| --- | --- | --- | --- | --- | --- |
| Log DMIC | 1 | **0.80** | 0.00 | 0.00 | 0.01 |
| Log H | **0.80** | 1 | 0.02 | 0.01 | 0.11 |
| Log Y | 0.00 | 0.02 | 1 | 0.78 | 0.26 |
| Log DBH | 0.00 | 0.01 | 0.78 | 1 | 0.64 |
| Log d | 0.01 | 0.11 | 0.26 | 0.64 | 1 |

The above results revealed that the best determination for Log DMIC variable is log mean height (Log H), suggesting a linear relationship between the log variables or a power law between DMIC and H.

Therefore, regression model for DMIC as a function of H is given by the following equation: DMIC = 0.0361 x H^2.2^. The F-statistic is 28 on 1 and 7 degree of freedom (DF) with p-value of 0.001, indicating the H variable significantly explains the DMIC. Due to the initial log transformation of the observation values and the distribution nature of the standard errors of log-normal, the mean values of the regression were recalculated according to Fergusson^8^ which helps to predict the bias when using a regression fit on log values.

Accordingly, DMIC biomass for YC stand (H=14 m) is calculated to be 12.1 t ha^-1^ with its maximum standard deviation (SD) of 2.28 t ha^-1^. The relative abundance of dead needles versus dead branches (1.4) was calculated according to literature^7^ using the attached DBH values of the YC stand.

Using the same stand as Miyaura and Hozumi^3^ 17 years later, Yoshida and Hijii^7^ suggested that even if the amount of in-crown dead leaves increases with age, the trajectory of their increments as a function of tree size may change from an exponential curve to a “saturation” curve with an increasing stand age and a decreasing slope. Nevertheless the slope of the mean curve is probably not significantly affected by height values of stands before or close to canopy closure, which is the typical case for YC.

Consequently, the approach used for YC couldn’t be directly applied to Matured Cedar (MC) stand for which the height is above 20 m. If this approach was applied to MC, H=22.5 m, the DMIC biomass would be 33.7 t/ha, i.e. 22 % higher than the living needles biomass.

**DMIC calculation for MC stand**

Keeping in mind that the DMIC values in the stand used by Yoshida and Hijii^7^ are probably maximum, the amount of dead organs (branches and needles) in the MC stand were calculated with the allometric equations published by these authors (W = a (D²)^b^, where W is the dry biomass in g, D is the DBH in cm, a = 95.7 and b = 0.728 for dead needles, a = 16.1 and b = 0.970 for dead branches).

The stand of Yoshida and Hijii^7^ was chosen purposely because the dendrometric characteristics are very similar to those of the MC stand. Such as:

- The mean height of trees and age are comparable for MC (22.5 vs 20 m, 33 years)
- The value of crown length (including dead branches) for MC is close (7 m approx.) to the one for 20 m height trees (7 m)^7^

**DMIC turnover in crown**

The Supplementary Table 3 displays the values of the DMIC/Alive materials in crown (branches + needles) ratios and the DMIC turnover calculated for the MC and YC stands.

We observed that the DMIC turnover values calculated for our stands are comparable with those already reported in literature even if two different methods were used for the calculation.

Supplementary Table 3: Calculated values of DMIC, ratios of DMIC versus alive material and turnover of DMIC in crown

| Reference | Dead Material In Crown  (DMIC, t/ha) | Ratio DMIC/Alive materials in crown | DMIC Turnover  (/year) |
| --- | --- | --- | --- |
| Katsuno *et al.*^2^ | 7.4 | - | 0.51 |
| Miyaura and Hozumi^3^ | 3.6 | 0.25 ^a,b^ | 0.13 ^a^ |
| Katagiri *et al.*^5^ | 9.5 | 0.24 | 0.46 |
| Kaneko *et al.*^6^ | 5.5 | 0.19 | 0.25 ^c^ - 1.9 ^a^ |
| Yoshida and Hijii^7^ | 34.1 | 0.95-1.22 | 0.17 |
| **This study, MC** | **23.0** | **0.45** | **0.35** |
| **This study, YC** | **12.1** | **0.21** | **0.88** |

^a^ only needles taken into account; ^b^ derived from the publication; ^c^ only branches taken into account

**References**

**1.** Saito, H. & Shidei, T. Studies on the productivity and its estimation methodology in a young stand of *Cryptomeria japonica D. Don*. *Journal of Japanese Forestry Society* **67**, 52–62 (1973).

**2.** Katsuno, M., Hagihara, A. & Hozumi, K. Litterfall of a Japanese cedar (*Cryptomeria japonica*) stand (in Japanese). *Trans Annu Mtg Jpn For Soc* **95**, 363–364 (1984).

**3.** Miyaura, T. & Hozumi, K. Measurement of litterfall in a Sugi (*Cryptomeria japonica*) plantation by the cloth-trap method. *J Jpn For Soc* **71**, 69–73 (1989).

**4.** Tange, T., Suzuki, M., Negisi, K. & Suzuki, S. Differences in the amount of dead branches and leaf material in young Cryptomeria japonica stands in relation to spacing. *The Japanese Journal of Ecology* **39**, 139–146 (1989).

**5.** Katagiri, S., Kaneko, N. & Obatake, Y. Nutrient cycling in a Sugi *(Cryptomeria japonica D. Don)* stand with insufficient management: nutrient accumulation in aboveground and soil and nutrient return by litterfall and rainfall (in Japanese with English Summary). *Bull Fac Agr Shimane Univ* **24**, 21–27 (1990).

**6.** Kaneko, N., Katagiri, S., Yamashita, H., Kitaoka, N. & Tominaga, A. A long term observation of litterfall of Japanese red cedar in Sanbe Experimental Forest of Shimane University (in Japanese with English summary). *Bull Fac Life Env Sci Shimane Univ* **2**, 7–13 (1997).

**7.** Yoshida, T. & Hijii, N. Spatiotemporal distribution of aboveground litter in a *Cryptomeria japonica* plantation. *Journal of Forest Research* **11**, 419-426 (2006).

**8.** Ferguson, R. River loads underestimated by rating curves *Water Resources Research* **22**, 74-76 (1986).
